# An unusual case of pulmonary granulocytic sarcoma treated with combined chemotherapy and radiation

**DOI:** 10.3332/ecancer.2013.368

**Published:** 2013-10-29

**Authors:** Vishwanath Sathyanarayanan, Nagesh Taterao Sirsath, Umesh Das, M Malathi, Suma Lakshmipathi Reddy, Kavitha S Srivatsa, Avinash Thumallapalli, L Appaji, BS Aruna Kumari

**Affiliations:** 1 Department of Medical Oncology, Kidwai Memorial Institute of Oncology, Bangalore, Karnataka 560029, India; 2 Department of Pathology, Kidwai Memorial Institute of Oncology, Bangalore, Karnataka 560029, India; 3 Department of Pediatric Oncology, Kidwai Memorial Institute of Oncology, Bangalore, Karnataka 560029, India

**Keywords:** granulocytic sarcoma, pulmonary, chemotherapy, radiation

## Abstract

We report an unusual case of a 6-year-old male child who presented with fever and a cough of one month’s duration. A bone marrow aspiration and cytogenetics were suggestive of acute myeloid leukaemia with t(8;21)(q22;q22). A chest x-ray and computed tomography of the thorax showed a soft tissue lesion in the right lung. The fine needle aspiration cytology (FNAC) of this lesion was suggestive of pulmonary granulocytic sarcoma. The patient was successfully treated with induction chemotherapy (cytosine arabinoside + daunomycin), followed by consolidation with high-dose cytosine arabinoside. In view of the persistent lesion in the right lung, the patient was given external beam radiotherapy (EBRT), which resulted in near total resolution of the lung granulocytic sarcoma. We report this case in view of its rarity and clinical importance, and to highlight the treatment options in this scenario.

## Case report

**Figure 1.**
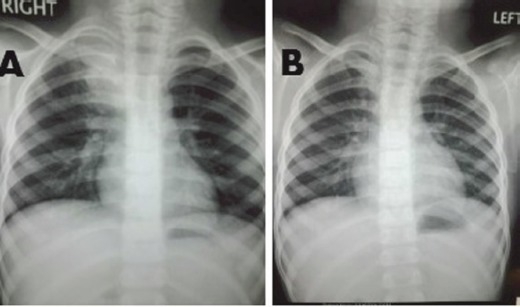
Panel A: Chest x-ray showing inhomogeneous opacity in the right upper lobe of lung. Panel B: Resolution of the inhomogeneous opacity following chemotherapy and radiation.

**Figure 2.**
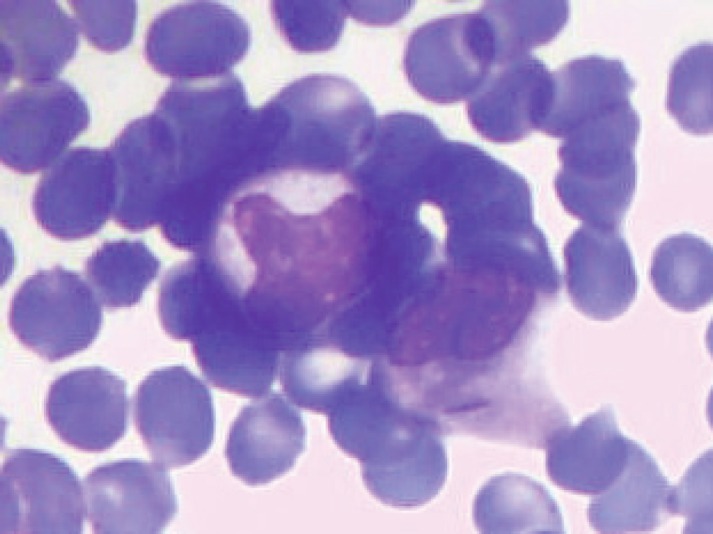
FNAC right lung lesion showing myeloblasts with auer rods suggestive of granulocytic sarcoma right lung.

**Figure 3.**
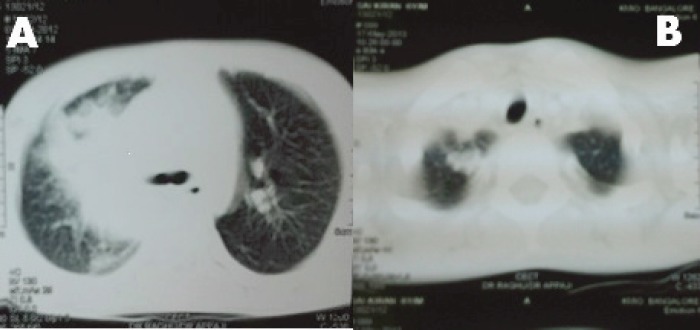
Panel A: computed tomography of thorax shows a soft tissue density lesion in the right lung measuring with a pleural-based nodule in the apical basal segment of right lower lobe. Panel B: computed tomography of thorax shows near total resolution of the soft tissue density lesion following chemotherapy and radiation.

A 6-year-old male child presented with high-grade fever and a cough with mucoid sputum of one month’s duration. There was no history of bleeding manifestations or chest pain or dyspnoea. He was the product of a non-consanguinous marriage. His birth, family, and developmental history were unremarkable. On examination, his weight was 14 kg and height 102 cm. He was febrile (temperature, 102 °F) with a pulse rate of 102 beats per minute. He was pale, and there was no evidence of peripheral palpable lymphadenopathy. Examination of the abdomen showed a palpable liver (2 cm below right costal margin) with no splenic enlargement. The rest of the systemic examination was normal. On evaluation, his haemogram showed a haemoglobin of 5.7 g%, white blood count of 7,700/cm^3^, absolute neutrophil count of 3,900 cm^3^, and platelet count of 34,000, with no blasts in the peripheral smear. The serum comprehensive metabolic panel, including random blood sugar, renal function tests, and liver function tests, was unremarkable. The serum lactate dehydrogenase level was 304 U/L and uric acid 3.2 mg/dl. Serum for HIV, HBsAg, and HCV were negative. The ECG and echocardiogram were within normal limits. The bone marrow aspiration was hypercellular with myeloblasts 40% and auer rods suggestive of acute myeloid leukaemia (AML). Cytogenetics showed t(8;21) (q22,q22). The chest x-ray showed an inhomogeneous opacity seen in the apex of right lung. (Figure 1, Panel A). The computed tomography of the thorax showed a heterogeneously enhancing soft tissue density lesion in the right lung measuring 5.8 × 5.7 cm. The lesion extended from the first to the third thoracic vertebrae, medially right bronchus, partially encasing the trachea and laterally extending up to the lateral chest wall. Also a nodule measuring 1.3 × 0.9 cm was seen in the posterior segment of the right upper lobe with a pleural-based nodule in the apical basal segment of right lower lobe (Figure 3). A right lung FNAC showed numerous alveolar macrophages and histiocytes with myeloblasts and auer rods suggestive of granulocytic sarcoma right lung (Figure 2). He was started on induction with cytosine arabinoside (100 mg/m^2^/day) × 7 days and daunomycin (45 mg/m^2^/day) × 3 days. As the bone marrow was in remission following induction, high-dose cytosine arabinoside (6 gm/m^2^/day) days 1–3) q 28 days × 4 cycles was given. In view of a persistent right upper lobe lung granulocytic sarcoma following consolidation chemotherapy, the patient was also given external beam radiotherapy (EBRT) to the chest – 24 Gy/16 fractions. Following chemotherapy and radiotherapy, the chest x-ray showed near total resolution of the lesion (Figure 1, Panel B), and the computed tomography of the chest showed regression of the lesion with residual sub-centimetric pleural-based nodules (Figure 3, Panel B).

## Discussion

Granulocytic sarcoma, also called chloroma, extramedullary leukaemia, myeloid sarcoma (MS), or myeloblastoma, is an unusual extramedullary tumour of immature myeloid cells [1]. It was initially described in the early 19th century, and later in 1853, King coined the word ‘chloroma’ in view of the green colour produced by myeloperoxidase [2].

Granulocytic sarcoma is seen in around 5% of patients with AML. Its incidence is increased in AML with t(8; 21) (q22;q22), FAB M4/M5 sub-types, infantile leukaemia and allogenic stem cell transplantation [3]. Other cytogenetic abnormalities associated with this entity include inversion 16 and 11 q abnormalities. In our case, it was AML with t(8;21)(q22;q22).

It can either precede, occur concomitantly, or follow the onset of acute and chronic leukaemias, usually acute myelogenous leukaemias. In this case report, a 6-year-old child had granulocytic sarcoma with AML as presentation. It is occasionally seen with myelodysplastic syndrome and myeloproliferative disorders [4].

The pathogenesis of this entity is poorly understood and reduced efficacy of cytotoxic agents and graft-versus-leukaemia response contribute to the aetiopathogenesis. Moreover other mechanisms involving adhesion and invasion of leukaemic blasts have also been suggested [5].

It commonly involves the bone, periosteum, soft tissue, lymph nodes, and skin. The involvement of other organs is rare and is reported in small case series/anecdotal case reports. The involvement of the gastrointestinal tract, head and neck region, central nervous system (CNS), pericardium, bronchus, kidney, bladder, testicles prostate, ovaries, uterus, and mediastinum has been reported [4, 6, 7]. The incidence of granulocytic sarcomas in each site is difficult to assess in view of its rarity; however, certain sites are more common, such as bones and periosteum, as they are close to the bone marrow. However, the incidence of certain sites varies**—**the head and neck region varies from 12% to 50% and gastrointestinal tract involvement is around 7% [4]. Rarely, it can involve the lung/pleura with only a handful of cases reported in the literature so far [8]. To mention a few, Kim *et al *in 2009 [9] reported a case of intrathoracic granulocytic sarcoma in a 37-year-old man following remission of four years of acute myeloid leukaemia. The chest computed tomography showed a large lung mass within the right middle lobe and hilar and mediastinal lymphadenopathy. The histopathological specimen showed leukaemic blasts suggestive of granulocytic sarcoma. Guimaraes in 2013 [10] reported a case of pulmonary granulocytic sarcoma mimicking an opportunistic infection in a patient with acute myeloid leukaemia. Radiological findings include lung mass or cavitation with or without associated lymphadenopathy. In our patient, a computed tomography of the thorax showed a heterogeneously enhancing soft tissue density lesion in the right lung with pleural-based nodule.

Myeloid granulocytic sarcoma poses a significant diagnostic challenge. Biopsy and staining with immunohistochemical markers play a vital role. The immunophenotype is characteristic based on whether myeloid sarcoma is granulocytic (MPO+, lysozymes+, CD34+/−), monoblastic (MPO−, CD68+, Lysozyme−, CD34+), myelomonoblastic (MPO+/−, CD68+, lysozyme+/−, CD34+/−), megakaryoblastic (Factor VIII+, CD31+), or erythroblastic variant (glycoprotein c+) [11]. Myeloid granulocytic sarcoma has to be differentiated from medium sized or large cell non-Hodgkin’s lymphoma. In addition, differentiation of granulocytic sarcomas from small round cell tumours is also critical [11, 12]. Tissue samples can also be sent for fluorescence *in situ *hybridisation and molecular analysis. Recent systematic fluorescence *in situ *hybridisation analysis on granulocytic sarcoma samples detected several chromosomal aberrations, including monosomy 7, trisomy 4, trisomy 8, trisomy 11, del(5q), and del(20q) [13]. The most frequent associated chromosomal abnormality is t(8;21), which our patient did have. However, it is often difficult to obtain sufficient tissue for such studies. In our patient, we did a FNAC that showed myeloblasts and auer rods suggestive of granulocytic sarcoma right lung.

Most guidelines suggest that patients with granulocytic sarcoma should be given the induction regimen as given for AML followed by consolidation chemotherapy regimen with high-dose cytosine arabinoside [11]. The role of surgical debulking is mainly to relieve compression over adjacent structures. Radiation therapy (RT) has been used to give rapid relief of symptoms and persistent disease following chemotherapy. The recommended dose of radiation is 20 Gy in 10–12 fractions [8].

Other modalities, such as the use of high-dose methylprednisolone, alpha-interferon, methotrexate, allogeneic bone marrow transplant, and autologous stem cell transplantation, have been used with variable success [8].

## Conclusion

We treated a 6-year-old male child with induction chemotherapy (cytosine arabinoside + daunomycin 7 + 3 regimen) followed by four cycles of high-dose cytosine arabinoside four weeks apart. In view of significant persistent granulocytic sarcoma deposits in the right lung, we decided to give EBRT to the chest – 24 Gy/16 fractions, which resulted in a significant reduction in the right lung mass.

Use of multimodality treatment strategies, including chemotherapy, radiation, and surgery, has shown long-term remissions in certain case series. However, larger studies are required to formulate guidelines.
